# Cell-type- and locus-specific epigenetic editing of memory expression

**DOI:** 10.1038/s41588-025-02368-y

**Published:** 2025-10-29

**Authors:** Davide M. Coda, Lisa Watt, Liliane Glauser, Mykhailo Y. Batiuk, Allison M. Burns, Cora L. Stahl, Lok Y. Wong, Johannes Gräff

**Affiliations:** 1 Laboratory of Neuroepigenetics, Brain Mind Institute, School of Life Sciences, École Polytechnique Fédérale de Lausanne (EPFL), Lausanne, Switzerland; 2 Bioinformatics Competence Center, École Polytechnique Fédérale de Lausanne (EPFL), Lausanne, Switzerland; 3 Synapsy Research Center for Neuroscience and Mental Health Research, École Polytechnique Fédérale de Lausanne (EPFL), Lausanne, Switzerland; 4 Present Address: Novartis Institutes for Biomedical Research, Basel, Switzerland; 5 Present Address: University of Toronto, Toronto, Ontario Canada

**Keywords:** Neuroscience, Epigenetics, Behavioural genetics

## Abstract

Epigenetic mechanisms have long been proposed to act as molecular mnemonics^1–3^, but whether the epigenetic makeup of a single genomic site can guide learnt behaviors remains unknown. Here we combined CRISPR-based epigenetic editing tools^4,5^ with c-Fos-driven engram technologies^6,7^ to address this question in memory-bearing neuronal ensembles. Focusing on the promoter of *Arc*, which encodes a master regulator of synaptic plasticity^8^, we found that its locus-specific and temporally controlled epigenetic editing is necessary and sufficient to regulate memory expression. Such effects occurred irrespective of the memory phase—during the initially labile period after learning and for fully consolidated memories—and were reversible within subject, testifying to their inherent plasticity. These findings provide a proof-of-principle that site-specific epigenetic dynamics are causally implicated in memory expression.

## Abstract (web summary)

CRISPR-based epigenetic editing is used in a cell-type-specific, locus-restricted and temporally controllable manner in the adult mouse brain to modulate memory expression.

## Main

Over the past two decades, several studies have indicated that epigenetic mechanisms—mainly histone acetylation and DNA methylation—may contribute to memory formation, storage and change^9^. Experimental support of this evidence has been obtained by their observation in whole-tissue homogenates or broadly defined cell types such as excitatory neurons following learning^10^, or by wide-range pharmacological agents^11^ and genetic approaches that modify epigenetic enzymes and, concomitantly, memory performance^12–14^. However, a causal interrogation of site-specific epigenetic modifications for regulating memory expression has thus far been lacking.

In recent years, accumulating evidence has shown that memories are in part encoded in sparse populations of defined brain cells, so-called engrams^15^. Engram cells are nowadays the closest approximation of a memory trace, that is, the physical substrate of a memory: activated by learning, they display enhanced structural and synaptic plasticity^16^, regulate memory retrieval^7^ and are of lasting nature^17^. Following learning, engram cells also display epigenetic modifications^18^, positioning this cellular subpopulation as an ideal place to query their mnemonic role in cell-type- and locus-resolved manner.

## Results

**Extended Data Fig. 1:**
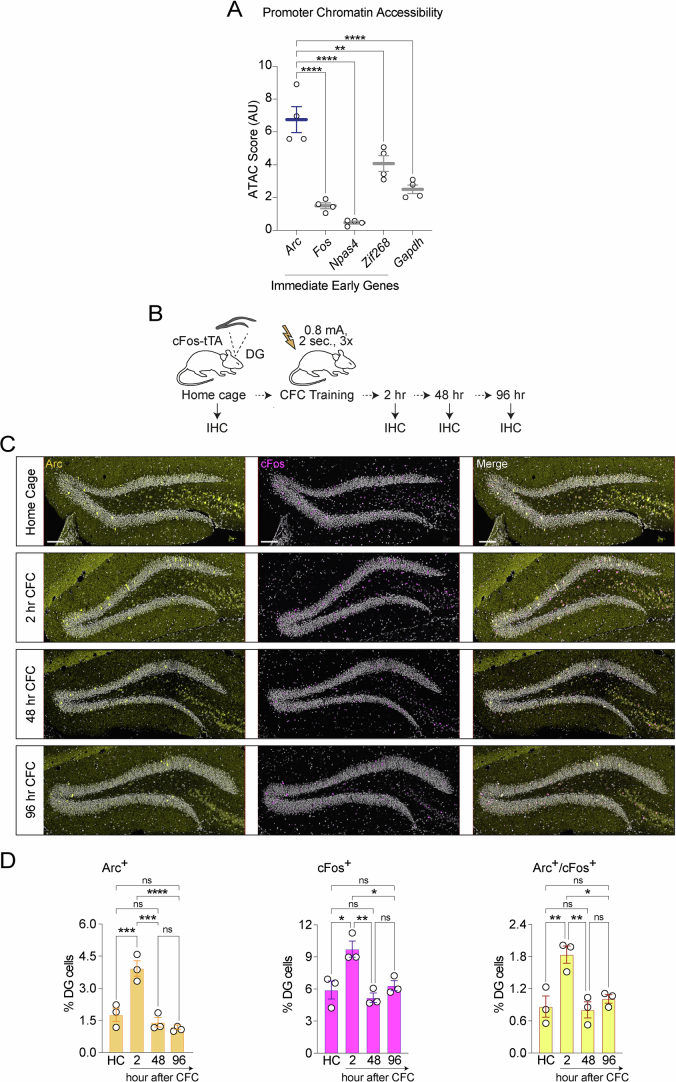
*Arc* promoter in dentate gyrus cells shows increased chromatin accessibility. (**A**) For dentate gyrus (DG) cells from home cage mice, plotted are the RPKM values for the ATAC-seq peaks detected at the promoter regions of representative immediate early genes (*Arc, Fos, Npas4, Zif268*) and housekeeping gene (*Gapdh*). Note that the *Arc* promoter displays an highly open chromatin conformation in the baseline state compared to other immediate early genes. Data are means ± SEM of three independent experiments compared by one-way ANOVA with Tukey’s post hoc test (**P < 0.01 and ****P < 0.0001). Data were obtained from Marco et al.^7^, GSE152954_ATAC_Desq2_Matrix_all_groups_RPKM.txt.gz. (**B**) Schematic of the time-course experiment to quantify the number of DG cells expressing Arc and cFos protein levels after CFC training in c-Fos-tTA mice. IHC, immunohistochemistry. (**C**) Representative confocal images showing protein expression levels of Arc (yellow) and cFos (magenta) in the DG of mice treated as described in A. Scale bars, 100 μm. (**D**) Quantification of the IHC analysis (n = 3), including number of DG cells positive for either Arc, cFos or both Arc and cFos. Data are means ± SEM compared by one-way ANOVA with Tukey’s multiple comparisons test. ns, not significant, *P ≤ 0.05, **P < 0.01, ***P < 0.001 and ****P < 0.0001. For all figure panels, see Supplementary Table 3 for summary statistics and p-values.

To do so, we developed CRISPR–dCas9-based epigenetic editing tools^4,5,19^ for cFos-driven engram tagging technologies^6^ that allow for the induction of locus-specific chromatin alterations within sparse memory-bearing neuronal ensembles. As editing target, we focused on *Arc*, an immediate early gene (IEG) known for its role in learning and synaptic plasticity^8,20^, whose promoter region is primed, that is, shows increased chromatin accessibility compared to other IEGs in the mouse dentate gyrus (DG) (Extended Data Fig. 1a–d). Since primed chromatin is a more dynamic epigenetic state than closed chromatin^21^, the *Arc* promoter represents an ideal site to probe for the importance of locus-specific epigenetic regulation.

**Fig. 1:**
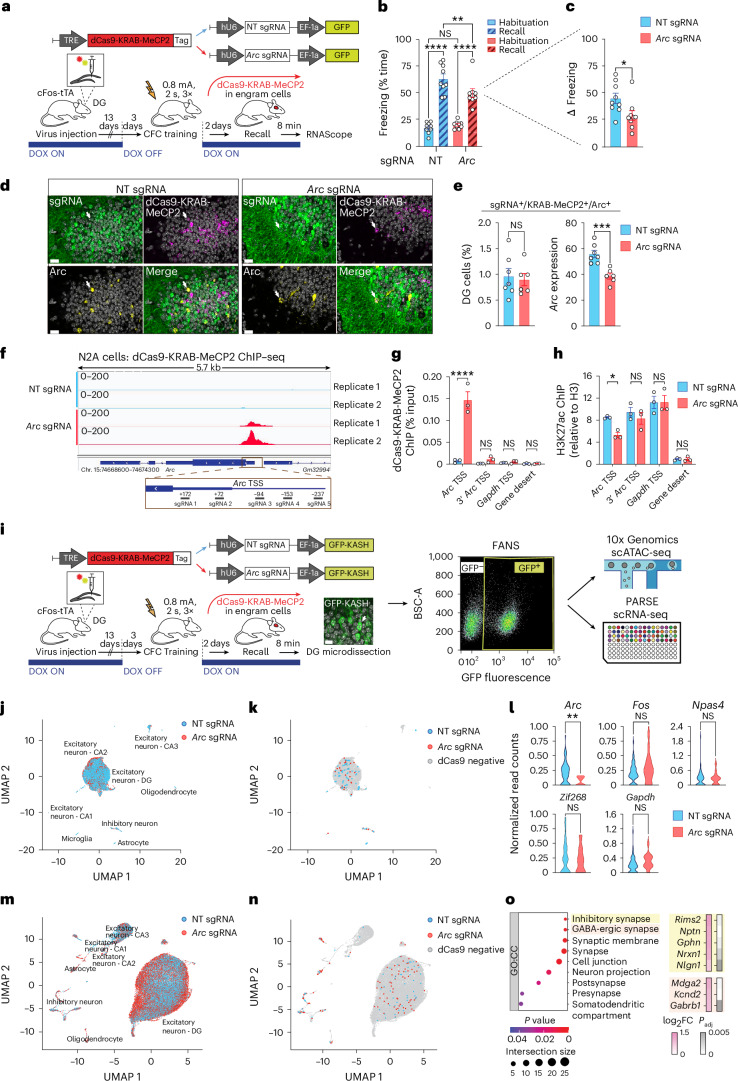
Epigenetically repressing memory formation. **a**, Schematic (top) and experimental design (bottom) for dCas9-KRAB-MeCP2-based epigenetic editing of the *Arc* promoter in cFos-tTA mice. For abbreviations, see text. **b**,**c**, Compared to animals injected with NT sgRNA (*n* = 9), dCas9-KRAB-MeCP2 animals injected with *Arc* sgRNA (*n* = 9) showed impaired memory formation. Data are means ± s.e.m. compared either by two-way ANOVA with Sidak’s and Fisher’s post hoc tests (**b**), or by a one-tailed, unpaired *t*-test (**c**). **d**, Representative confocal images showing mRNA expression levels of *Arc* (yellow) and dCas9-KRAB-MeCP2 (magenta) alongside with NT or *Arc* sgRNA (GFP, green) in the DG. Arrows indicate neurons positive for dCas9-KRAB-MeCP2, sgRNA and *Arc*. Scale bars, 20 μm. **e**, Quantification of the number of DG cells triple-positive for dCas9-KRAB-MeCP2^+^/sgRNA^+^/*Arc*^+^ and the level of *Arc* expression in the *Arc* sgRNA (*n* = 7) and NT sgRNA group (*n* = 6). Note that the dCas9-KRAB-MeCP2 manipulation does not result in a complete ablation of *Arc* expression in the DG. Data are means ± s.e.m. compared by a two-tailed, unpaired *t*-test. **f**, Top: Integrative Genomics Viewer genome browser of the *Arc* locus displaying ChIP–seq tracks for dCas9-KRAB-MeCP2 from N2A cells cotransfected with −*Arc* or NT sgRNA in two independent biological replicates. Bottom: enlarged view of the region surrounding the *Arc* promoter with the genomic sequences targeted by the sgRNAs highlighted. **g**,**h**, ChIP–qPCR on N2A cells treated as in **f** showing occupancy of dCas9-KRAB-MeCP2 (**g**) and H3K27ac (**h**) at the TSS of *Arc*. Plotted are means ± s.e.m. of three independent experiments compared by two-way ANOVA with Sidak’s multiple comparisons test. **i**, Experimental workflow to profile dCas9-mediated epigenetic and transcriptional changes in vivo by scATAC-seq and scRNA-seq. cFos-tTA mice were treated as described in **a**, except that in the sgRNA constructs nuclear GFP-KASH was expressed instead of GFP. A representative confocal image confirming GFP-KASH localization to the nuclear membrane in the DG is shown, alongside with a representative FANS plot showing a clear separation between the GFP-KASH- and GFP-KASH^+^ nuclei populations. Scale bar, 10 μm. **j**, UMAP of 9,452 nuclei from the DG of mice described in **i** colored according to their assignment to the *Arc* or NT sgRNA group. For each cluster, the corresponding cell type is annotated. **k**, As in **j**, except that dCas9-positive nuclei are displayed instead. **l**, For the nuclei identified in **k**, the normalized read counts for the ATAC-seq peaks detected at the promoter regions of *Arc* and other IEG or control genes (*Fos*, *Npas4*, *Zif268*, *Gapdh*) are plotted. Data are compared by a two-tailed, unpaired *t*-test. **m**, UMAP of 82,841 nuclei from the DG of mice described in **i** colored according to their assignment to the *Arc* or NT sgRNA group. For each cluster, the corresponding cell type is annotated. **n**, As in **m**, except that dCas9-positive nuclei are displayed instead. **o**, Functional enrichment analysis of genes differentially expressed in dCas9-positive excitatory neurons from either *Arc* or NT sgRNA mice. Left: the top gene ontology (GO) terms (cellular compartment (CC)) are displayed. Right: for the top two GO categories, the most strongly regulated genes are listed alongside their fold change value (log_2_) in the *Arc* sgRNA sample compared to the NT sgRNA sample, and matched adjusted P value (*P*_adj_). For all panels: NS, not significant; *P < 0.05, **P < 0.01 and ****P < 0.0001. See Supplementary Table 3 for summary statistics and *P* values.

To assess the behavioral consequences of epigenetically editing the *Arc* promoter within engram cells, we first engineered the epigenetic repressor dCas9-KRAB-MeCP2 (ref. ^5^) in a spatiotemporally regulatable manner, namely by combining it with an OFF doxycycline (DOX)-controllable tetracycline-responsive element (TRE) in a lentiviral construct (Fig. 1a). We stereotaxically delivered this construct into the DG of cFos-tTA mice, which express the tetracycline-controlled transactivator (tTA) upon learning^6^. We used a second lentivirus expressing five U6-driven single-guide RNAs (sgRNAs) targeting the *Arc* promoter, or as controls U6-driven nontargeting (NT) sgRNAs.

**Extended Data Fig. 2:**
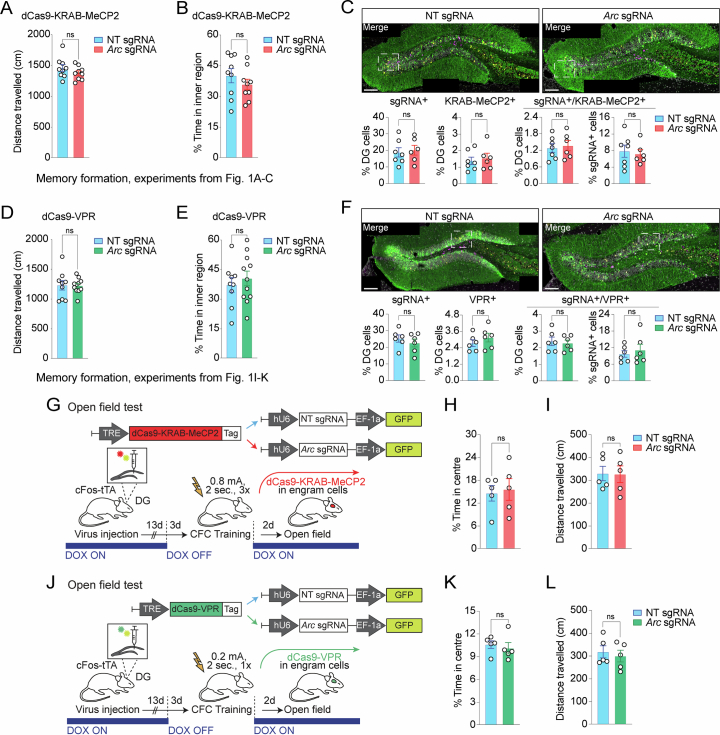
In vivo CRISPR-dCas9-based epigenetic editing of the *Arc* promoter does not impact basal locomotion and anxiety levels. (**A-B**) For mice from the dCas9-KRAB-MeCP2 experiment of Fig. 1a–c, displayed are (A) the average distance travelled and (B) the time spent in the inner region of the conditioning chamber during the three minutes habituation phase of the behavioural conditioning. For both the NT or *Arc* sgRNA groups, n = 9. (**C**) Top panel: for the dCas9-KRAB-MeCP2 experiment of Fig. 1a–c, confocal images showing expression levels of *Arc* mRNA (yellow) and dCas9-KRAB-MeCP2 mRNA (magenta) alongside with NT or *Arc* sgRNA (GFP, green) in the dentate gyrus (DG). Dotted squares delimitate the areas corresponding to the enlarged images in Fig.1d. Scale bars, 100 μm. Bottom panel: quantification of C. For the NT or *Arc* sgRNA groups, n = 7 and 6, respectively. (**D-E**) As in A-B, but for the dCas9-VPR experiment of Fig. 2a–c. For the NT or *Arc* sgRNA groups, n = 9 and 11, respectively. (**F**) As in C, but for the dCas9-VPR experiment of Fig. 2a–c. For both the NT or *Arc* sgRNA groups, n = 6. (**G**) Schematic of the dCas9-KRAB-MeCP2 open field experiment: mice were treated as in Fig. 1a, except that an open field test was performed two days after the CFC training. (**H-I**) For mice expressing the dCas9-KRAB-MeCP2 with the NT (n = 5) or *Arc* sgRNA (n = 5), displayed are (H) the time spent in the central area of the open field arena and (I) the average distance travelled during the test. Data were compared by a two-tailed, unpaired t test (ns, not significant) and plotted as means ± SEM. (**J-L**) As in G-I, except that dCas9-VPR is expressed instead. For both the NT or *Arc* sgRNA groups, n = 5. All data were compared by a two-tailed, unpaired t tests (ns, not significant) and plotted as means ± SEM. For all figure panels, see Supplementary Table 3 for summary statistics and p-values.

To trigger dCas9-KRAB-MeCP2 expression in engram cells activated by learning, cFos-tTA mice were taken off DOX 3 days before contextual fear conditioning (CFC), an associative memory task in which mice learn to pair a specific context with an unpleasant experience (that is, an electrical footshock). Immediately after CFC, mice were put on DOX again to prevent learning-unrelated TRE expression. Two days later, we measured the animals’ memory performance by re-exposing them to the conditioned context in the absence of footshock (Fig. 1a). We found that dCas9-KRAB-MeCP2 plus *Arc* sgRNA mice exhibited significantly less freezing (that is, the learnt association between footshock and context) than mice expressing NT sgRNA (Fig. 1b), indicating reduced memory formation. Normalizing the freezing rates during the recall phase to those during preconditioning (∆ freezing) confirmed that the reduction in memory expression was not due to alterations in baseline freezing behavior (Fig. 1c). Moreover, measurements of locomotion (distance traveled) and anxiety (time spent in the inner region of the behavioral apparatus) did not differ between the two groups (Extended Data Fig. 2a,b,g–i), excluding memory-unrelated effects of the dCas9-KRAB-MeCP2 manipulation.

**Extended Data Fig. 3:**
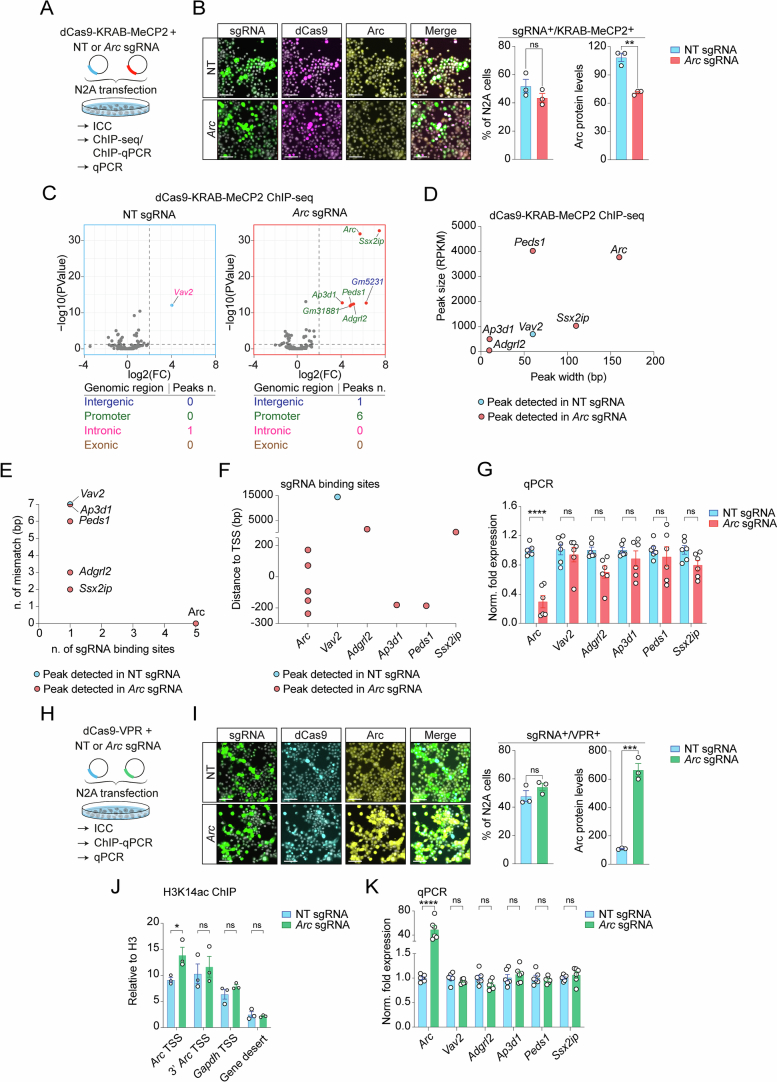
CRISPR-dCas9-based epigenetic editing enables the bidirectional control of *Arc* promoter activity with few off-target effects. (**A**) N2A cells were co-transfected with constructs for dCas9-KRAB-MeCP2 and NT or *Arc* sgRNA, and after 48 h collected for immunocytochemistry (ICC), ChIP-seq/ChIP-qPCR and qPCR analyses. (**B**) Left panel, representative ICC images revealing a high co-transduction efficiency of sgRNA (co-expressing GFP, green) and dCas9-KRAB-MeCP2 (FLAG-tagged, magenta), as well as a reduction in Arc protein levels (yellow) for the double positive cells in the *Arc* sgRNA sample. Cell nuclei are stained with DAPI. Scale bars, 50 μm. Right panel, Quantification of the ICC analysis, including number of N2A cells double positive for dCas9-KRAB-MeCP2 + /sgRNA+ and the level of Arc protein in these cells for the NT (n = 3) or *Arc* (n = 3) sgRNA samples. Data are means ± SEM of independent experiments compared by a two-tailed, unpaired t test. (**C**) For the samples described in Fig.1f, volcano plots showing genomic sites significantly enriched for dCas9-KRAB-MeCP2 chromatin binding (p-value < 0.05, fold change > 2, EdgeR differential enrichment analysis, see Methods). Tables below the graphs summarize the peaks’ features for each sample and dots in D are color-coded accordingly. One and 6 off-targets were identified for the NT sgRNA and *Arc* sgRNA, respectively. Of these sites, two of them were associated to predicted genes (*Gm5231* and *Gm31881*) whilst the other 5 were assigned to genes that either show low expression levels (according to the Allen Mouse Brain Atlas) in adult dentate gyrus (*Vav2*, *Adgrl2* and *Peds1*), or have not been implicated in fear memory processes (*Ap3d1*, *Ssx2ip*). The differential enrichment analysis results are reported in Supplementary Table 1. (**D-F**) For the dCas9-KRAB-MeCP2 binding sites described in C, plotted are: (D) the averaged ChIP-seq peak width and size; (E) the number of mismatches as predicted by the Cas-OFFinder sofware (y axes, in bp) and the number of sgRNA binding sites (x axes); (F) the position of each matching sgRNA relative to the TSS. Bp, base pair. Bp, base pair; RPKM, reads per kilobase per million mapped reads. (**G**) For the experiment described in A, q-PCR analysis was performed for the protein coding genes identified in C. dCas9-KRAB-MeCP2 plus *Arc* sgRNA downregulated *Arc* mRNA levels compared to the control condition with minimal off-target effects. Data are means ± SEM of three independent experiments compared by two-way ANOVA with Sidak’s multiple comparisons test. (**H**) As in A, except that dCas9-VPR is expressed instead. (**I**) Representative ICC images of N2A cells treated as described in H (left panel) and relative quantification (right panel). sgRNA, dCas9-VPR or Arc positive cells are in green, cyan or yellow, respectively. Cell nuclei are stained with DAPI. Scale bars, 50 um. Data are means ± SEM of three independent experiments compared by a two-tailed, unpaired t test. (**J**) ChIP-qPCR on N2A cells treated as in H showing occupancy of H3K14ac at the TSS of *Arc*. Plotted are means ± SEM of three independent experiments compared by two-way ANOVA with Sidak’s multiple comparisons test. (**K**) For the experiment described in H, q-PCR analysis was performed for the protein coding genes identified in C. dCas9-VPR plus *Arc* sgRNA upregulated *Arc* mRNA levels compared to the control condition with no off-target effects. Data are means ± SEM of three independent experiments compared by two-way ANOVA with Sidak’s multiple comparisons test. For all figure panels: ns, not significant; *P < 0.05 and ****P < 0.0001. For all figure panels, see Supplementary Table 3 for summary statistics and p-values.

At the molecular level, *Arc* expression in dCas9-KRAB-MeCP2-positive cells was reduced in the *Arc* sgRNA compared to the NT group, whereas the percentage of DG cells expressing both the dCas9-KRAB-MeCP2 and the sgRNA vector was similar between the two groups (Fig. 1d,e and Extended Data Fig. 2c). Epigenetically, dCas9-KRAB-MeCP2 binding to the *Arc* promoter decreased the occupancy of the transcription-associated chromatin mark H3K27ac, whereas the downregulation of *Arc* mRNA was paralleled by few off-target effects both in terms of sgRNA binding and ensuing expression changes (Fig. 1f–h and Extended Data Fig. 3a–g), as measured in N2A cells. In vivo, dCas9-KRAB-MeCP2 expression led to a closing of the *Arc* promoter as assessed by single-cell assay for transposase-accessible chromatin using sequencing (scATAC-seq) following fluorescence-activated nuclei sorting (FANS), and elicited downstream transcriptional changes of genes regulating inhibitory synaptic plasticity and neurotransmission (Fig. 1i–o).

**Fig. 2:**
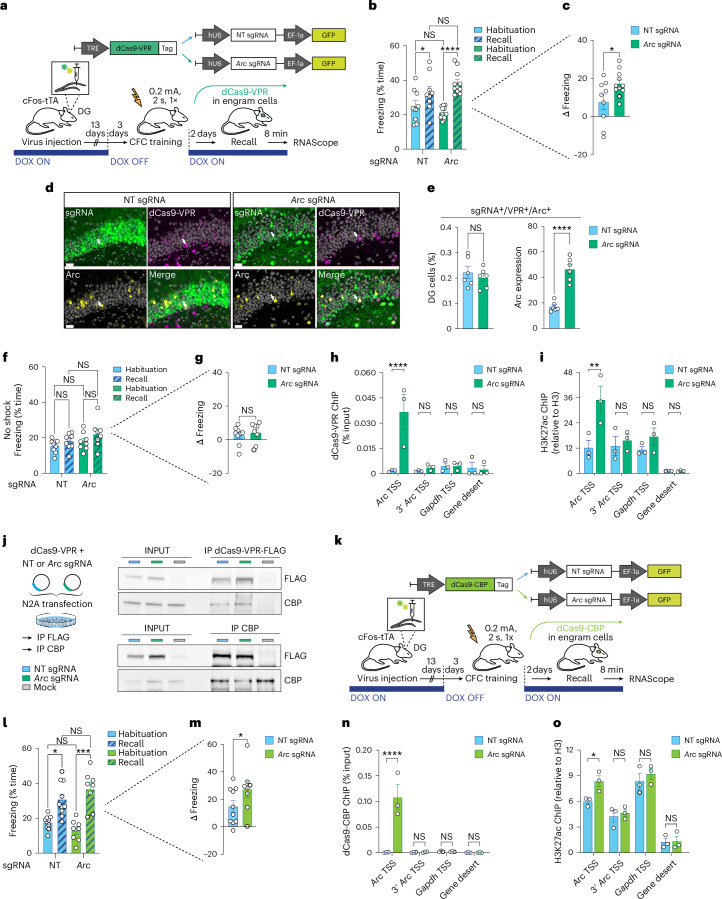
Epigenetically enhancing memory formation. **a**, Schematic (top) and experimental design (bottom) for dCas9-VPR-epigenetic editing of the *Arc* promoter in cFos-tTA mice. For abbreviations, see text. **b**,**c**, Compared to animals injected with NT sgRNA (*n* = 9), dCas9-VPR plus Arc sgRNA-injected animals (*n* = 11) showed improved memory recall. Data are means ± s.e.m. compared either by two-way ANOVA with Sidak’s and Fisher’s post hoc tests (**b**) or by a one-tailed, unpaired *t-*test (**c**). **d**, Representative confocal images showing mRNA expression levels of *Arc* (yellow) and dCas9-VPR (magenta) alongside with NT or *Arc* sgRNA (GFP, green) in the DG. Arrows indicate neurons positive for dCas9-VPR, sgRNA and *Arc*. Scale bars, 20 μm. **e**, Quantification of the number of DG cells triple-positive for dCas9-VPR^+^/sgRNA^+^/*Arc*^+^ and the level of *Arc* expression in the *Arc* sgRNA (*n* = 6) and NT sgRNA group (*n* = 6). Data are means ± s.e.m. compared by a two-tailed, unpaired *t*-test. **f**,**g**, As in **a**, except that no shock was administered during context exposure on the training day for mice injected with dCas9-VPR plus NT sgRNA (*n* = 9) or the *Arc* sgRNA (*n* = 8). Data are means ± s.e.m. compared either by two-way ANOVA with Sidak’s and Fisher’s post hoc test (**f**) or by a one-tailed, unpaired *t*-test (**g**). **h**,**i**, N2A cells were cotransfected with constructs for dCas9-VPR and *Arc* or NT sgRNA, and collected after 48 h. Displayed are ChIP–qPCR analyses showing occupancy of dCas9-VPR (**h**) and H3K27ac (**i**) at the TSS of *Arc*. For **h** and **i**, means ± s.e.m. of three independent experiments compared by two-way ANOVA with Sidak’s multiple comparisons test are plotted. **j**, As in **h**, except that cells were collected for co-IP analysis. Displayed are representative co-IP western blot images revealing that dCas9-VPR interacts with CBP, and vice versa. **k**, Schematic (top) and experimental design (bottom) for dCas9-CBP-based epigenetic editing of the *Arc* promoter in cFos-tTA mice. **l**,**m**, Compared to animals injected with NT sgRNA (n = 10), dCas9-CBP animals injected with *Arc* sgRNAs (*n* = 8) showed improved memory recall. Data are means ± s.e.m. compared either by two-way ANOVA with Sidak’s and Fisher’s post hoc tests (**l**), or by a one-tailed, unpaired *t*-test (**m**). **n**,**o**, As in **h** (**n**) and **i** (**o**), except that dCas9-CBP is expressed instead. For **n** and **o**, plotted are means ± s.e.m. of three independent experiments compared by two-way ANOVA with Sidak’s multiple comparisons test. For all panels: NS, not significant; *P < 0.05, **P < 0.01, ***P < 0.001 and ****P < 0.0001. See Supplementary Table 3 for summary statistics and *P* values. Source data

Next, we tested whether an epigenetic activation of *Arc* would lead to the opposite behavioral effect. To this end, we stereotaxically injected an OFF DOX-inducible version of the epigenetic activator dCas9-VPR^4,19^ (TRE-dCas9-VPR), as well as lentiviruses containing *Arc* or NT sgRNAs into the DG of cFos-tTA mice (Fig. 2a). We used the same experimental timeline as before, except for employing a subthreshold CFC protocol^22^, which led to a modest memory formation in the NT control group (Fig. 2b). In contrast, we found that dCas9-VPR plus *Arc* sgRNA mice showed a robust increase in freezing at recall, indicating improved memory formation (Fig. 2b,c). No differences in locomotion or baseline behavior were observed between the two groups (Extended Data Fig. 2d,e,j–l).

**Extended Data Fig. 4:**
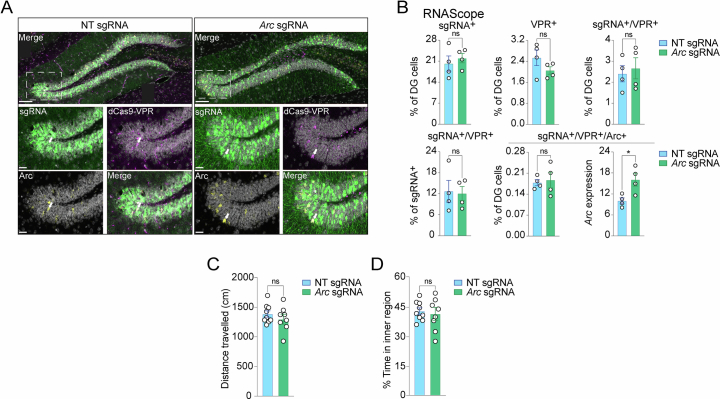
Context only control experiment: dCas9-VPR upregulates *Arc* in engram cells and does not impact basal locomotion and anxiety levels. (**A-B**) Brains from the mice of the dCas9-VPR experiment of Fig. 2f–g were analyzed by RNAScope. Displayed are (A) representative confocal images and relative quantification (B) showing mRNA expression levels of *Arc* (yellow) and dCas9-VPR (magenta) alongside with NT or *Arc* sgRNA (GFP, green) in the dentate gyrus (DG). Scale bars, 100 μm (overview) and 20 μm (zoom-in). Arrows indicate neurons positive for dCas9-VPR, sgRNA and Arc. Note that in these cells *Arc* signal is higher for the *Arc* sgRNA group of mice compared to the control NT sgRNA animals (n = 4 each). Data are means ± SEM compared by a two-tailed, unpaired t test. (**C-D**) For the same mice of Fig. 2f–g, displayed are (C) the average distance travelled and (D) the time spent in the inner region of the conditioning chamber during the three minutes habituation phase of the behavioral conditioning. For the NT or *Arc* sgRNA groups, n = 9 and 8, respectively. Data were compared by a two-tailed, unpaired t test (ns, not significant) and plotted as means ± SEM. For all figure panels, see Supplementary Table 3 for summary statistics and p-values.

**Extended Data Fig. 5:**
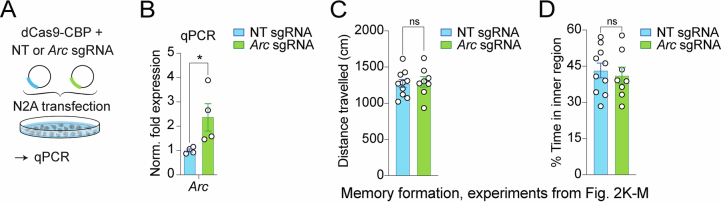
dCas9-CBP-mediated epigenetic activation of Arc in engram cells enhances memory formation. (**A**) N2A cells were co-transfected with constructs for dCas9-CBP and NT or *Arc* sgRNA, and after 48 h collected for qPCR analysis. (**B**) qPCR on N2A cells treated as in A revealing that dCas9-CBP binding to the *Arc* promoter upregulates *Arc* expression. Displayed are means ± SEM of four independent experiments compared by a two-tailed, unpaired t test. (**C-D**) For mice from the experiment described in Fig. 2k–m, displayed are (C) the average distance travelled and (D) the time spent in the inner region of the conditioning chamber during the three minutes habituation phase of the behavioral conditioning. For the NT or *Arc* sgRNA groups, n = 10 and 8, respectively. Data were compared by a two-tailed, unpaired-t test and plotted as means ± SEM. For all figure panels: ns, not significant and *P ≤ 0.05. For all figure panels, see Supplementary Table 3 for summary statistics and p-values.

Molecular analyses revealed similar infection efficiency, but higher levels of *Arc* in engram cells positive for dCas9-VPR and *Arc* sgRNA compared to NT controls (Fig. 2d,e and Extended Data Fig. 2f). Notably, context exposure without conditioning also led to increased *Arc* mRNA expression in dCas9-VPR plus *Arc* sgRNA mice but did not alter freezing levels, testifying to the specificity of the dCas9-VPR-based memory enhancement to the fearful experience (Fig. 2f,g and Extended Data Fig. 4a–d). Epigenetically, we found that dCas9-VPR binding to the *Arc* promoter increased H3K27ac, H3K14ac and *Arc* mRNA with no off-target effects (Fig. 2h,i and Extended Data Fig. 3h–k), which was likely mediated by a direct interaction with the histone acetyltransferase CREB-binding protein (CBP) (Fig. 2j). Accordingly, the expression of dCas9-CBP in DG engram cells was able to fully recapitulate the behavioral, epigenetic and transcriptional effects of dCas9-VPR (Fig. 2k–o and Extended Data Fig. 5a–d). Together with the dCas9-KRAB-MeCP2 experiments, these findings suggest that the epigenetic makeup of a single locus within sparse DG engram cells is necessary and sufficient to regulate memory expression.

**Fig. 3:**
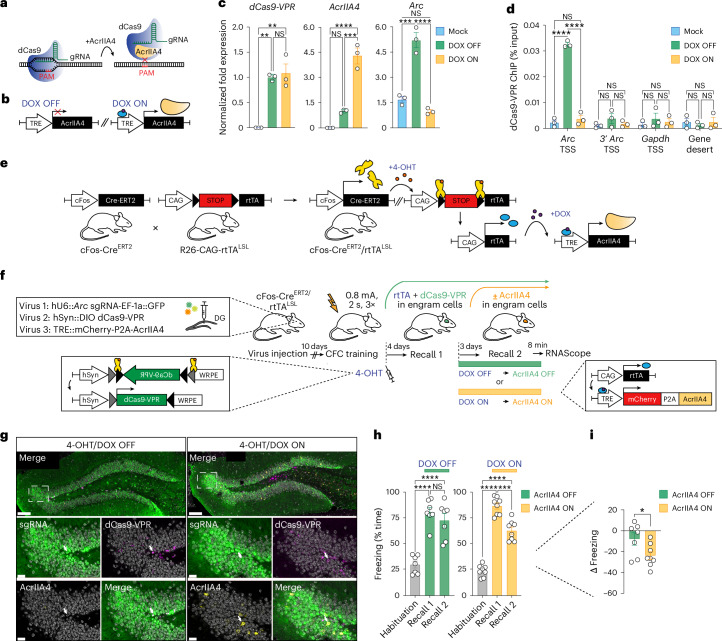
Within-subject reversible epigenetic memory editing. **a***,***b**, Schematic of the mechanism of action of the anti-CRISPR protein AcrIIA4 (**a**), which was rendered inducible by combining it with DOX ON activated TRE promoter (**b**). **c**, qPCR on N2A cells transfected with dCas9-VPR, *Arc* sgRNA and AcrIIA4 showing that AcrIIA4 induction abolishes the dCas9-VPR mediated increase in *Arc* mRNA. Data are means ± s.e.m. of three independent experiments compared by one-way ANOVA with Tukey’s post hoc test. **d**, ChIP–qPCR on N2A cells treated as in **c** showing occupancy of dCas9-VPR at the TSS of *Arc*. Plotted are means ± s.e.m. of three independent experiments compared by two-way ANOVA with Tukey’s multiple comparisons test. **e**, Breeding schematic of the new double transgenic mouse line cFos-Cre^ERT2^/rtTA^LSL^. **f**, Experimental timeline using the cFos-Cre^ERT2^/rtTA^LSL^ line to uncouple the expression of dCas9-VPR (4-OHT dependent) from the expression of AcrIIA4 (DOX ON-dependent). **g**, Representative confocal images showing the expression of dCas9-VPR mRNA (magenta) alongside with *Arc* sgRNA (GFP, green) and the mCherry mRNA reporter for AcrIIA4 (yellow) in the DG. Scale bars, 100 μm. Smaller panels show enlarged areas within the dotted squares. Scale bars, 20 μm. Arrows indicate neurons positive for either dCas9-VPR and *Arc* sgRNA (4-OHT/DOX OFF) or dCas9-VPR, *Arc* sgRNA and AcrIIA4 (4-OHT/DOX ON). **h**,**i**, Compared to animals expressing dCas9-VPR plus *Arc* sgRNA and kept off DOX (*n* = 7), animals put ON DOX after the first recall to induce AcrIIA4 (*n* = 8) showed impaired memory retention, expressed as freezing percentage (**h**) and as freezing differential between the second and first recall (Δ FreezingR = Recall 2 − Recall 1) (**i**). Data are means ± s.e.m. compared either by two-way ANOVA with Sidak’s post hoc test (**h**) or a one-tailed, unpaired *t*-test (**i**). For all panels: NS, not significant; *P < 0.05, **P < 0.01, ***P < 0.001 and ****P < 0.0001. See Supplementary Table 3 for summary statistics and *P* values.

**Extended Data Fig. 6:**
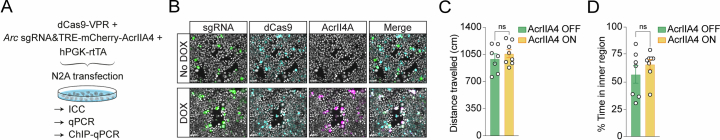
AcrIIA4-dependent rescue of dCas9-VPR mediated memory enhancement does not impact basal locomotion and anxiety levels. (**A**) Experimental design to test the effect of AcrIIA4 in vitro in the presence of dCas9-VPR and *Arc* sgRNA. 48 h after transfection, N2A cells were collected for immunocytochemistry (ICC), qPCR (Fig. 3c) and ChIP-qPCR (Fig. 3d). (**B**) Representative images of N2A cells from three independent experiments treated as described in A, showing DOX-inducible AcrIIA4 expression (mCherry reporter) and high co-transduction efficiency of *Arc* sgRNA (co-expressing GFP) and dCas9-VPR (FLAG-tagged). Cell nuclei are stained with DAPI. Scale bars, 50 μm. (**C-D**) For mice from the AcrIIA4 experiment of Fig. 3h–i, displayed is (C) the average distance travelled and (D) the time spent in the inner region of the conditioning chamber during the three minutes habituation phase of the behavioral conditioning. For the AcrIIA4 OFF or ON groups, n = 7 and 8, respectively. In C and D data were compared by a two-tailed, unpaired t test and plotted as means ± SEM (ns, not significant). DOX, doxycycline. For all figure panels, see Supplementary Table 3 for summary statistics and p-values.

Subsequently, we asked whether the epigenetic editing effects on behavior are reversible within subject, which serves the purpose to examine their plasticity, a defining feature of any memory-related process. To this end, we employed the anti-CRISPR protein AcrIIA4^23^, which functions by occluding the PAM recognition domain of dCas9, hence negating dCas9 binding to the DNA (Fig. 3a), and rendered it inducible by coupling it to a DOX ON controllable TRE promoter (Fig. 3b). In vitro, AcrIIA4 induction reverted the dCas9-VPR-mediated increase of *Arc* within 3 days (Fig. 3c,d and Extended Data Fig. 6a,b).

To use this setup in vivo, we designed a double transgenic labeling system that allows for the sequential induction of dCas9-VPR and AcrII4 in the same engram cells. Specifically, we crossed cFos-Cre^ERT2^ mice^11^, in which tamoxifen-dependent Cre-recombinase expression is restricted to engram cells, with R26-CAG-rtTA^LSL^ mice^24^, which contain a loxP-flanked reverse tetracycline-controlled transactivator (rtTA) that binds the TRE promoter in a DOX-ON-dependent manner (Fig. 3e). In the resulting double transgenic cFos-Cre^ERT2^/R26-CAG-rtTA^LSL^ mice (hereafter referred to as cFos-Cre^ERT2^/rtTA^LSL^), we stereotaxically delivered a Cre-dependent version of dCas9-VPR (using a double inverted open reading frame (DIO)-dCas9-VPR), AcrIIA4 under the control of TRE, and the *Arc* sgRNA to their DG (Fig. 3f). Immediately after CFC, cFos-Cre^ERT2^/rtTA^LSL^ mice received an injection of 4-hydroxytamoxifen (4-OHT), which triggered the expression of both rtTA and dCas9-VPR in learning-activated neurons. Four days later, memory formation was measured with a first recall session as before. Thereafter, one group of mice received DOX, which induced AcrIIA4 expression, and the other group of mice did not receive DOX, thus AcrIIA4 was not expressed (Fig. 3g). Another 3 days later, all mice underwent a second recall test, by which their memory retention could be assessed.

**Extended Data Fig. 7:**
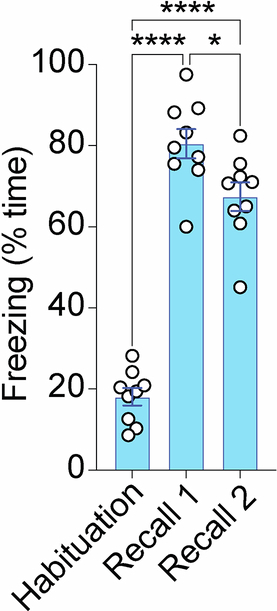
Memory retention naturally decreases over time. Freezing levels for cFos-Cre^ERT2^/rtTA^LSL^ mice (n = 9) that underwent the same CFC protocol described in Fig. 3 followed by two recall sessions, the first one 72 h after CFC and the second one on the following day. Note that animals spent significantly less time freezing at the Recall 2 timepoint compared to the Recall 1 timepoint. Data are means ± SEM compared by one-way ANOVA with Tukey’s post hoc test (*P < 0.05 and ****P < 0.0001). See Supplementary Table 3 for summary statistics and p-values.

For mice in which dCas9-VPR but not AcrIIA4 was induced (that is, the DOX OFF group), we found that freezing levels on the second recall did not differ significantly from those of the first recall (Fig. 3h). Since mice without dCas9-VPR induction normally show reduced freezing upon the second memory recall (Extended Data Fig. 7), this finding indicates that dCas9-VPR induction leads to improved memory retention, in line with dCas9-VPR facilitating memory formation (Fig. 2c). Conversely, for animals in which dCas9-VPR induction was followed by AcrIIA4 activation (the DOX ON group), we observed reduced freezing at the second recall (Fig. 3h,i), indicating that the improved memory retention upon dCas9-VPR was negated. Importantly, AcrIIA4 induction did not alter basal locomotion nor anxiety levels (Extended Data Fig. 6c,d). These reversibility experiments show that the observed behavioral effects are a direct consequence of the epigenetic regulation of *Arc*, which is thus plastic within the same animal.

**Fig. 4:**
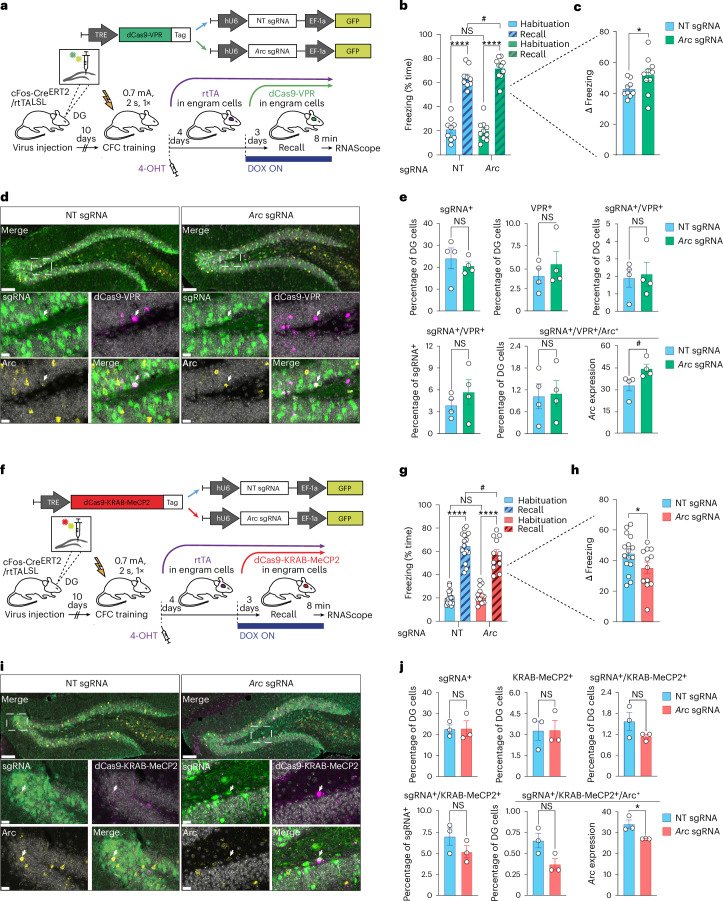
Epigenetic editing beyond memory malleability. **a**, Schematic (top) and experimental design (bottom) for inducible expression of the dCas9-VPR system in cFos-Cre^ERT2^/rtTA^LSL^ mice. For abbreviations, see text. **b**,**c**, Compared to animals injected with dCas9-VPR plus NT sgRNA (*n* = 9), animals injected with dCas9-VPR plus *Arc* sgRNA (*n* = 10) showed improved memory recall. Data are means ± s.e.m. compared either by two-way ANOVA with Sidak’s and Fisher’s post hoc tests (**b**) or by a one-tailed, unpaired *t*-test (**c**). **d**, Confocal images showing expression levels of *Arc* mRNA (yellow) and dCas9-VPR mRNA (magenta) alongside with NT or *Arc* sgRNA (GFP, green) in the DG. Scale bars, 100 μm. Smaller panels show enlarged areas within the dotted squares. Scale bars, 20 μm. Arrows indicate neurons positive for dCas9-VPR, sgRNA and *Arc*. **e**, Quantification of **d**, showing increased *Arc* expression in triple-positive neurons from the *Arc* sgRNA group compared to the NT sgRNA group (*n* = 4 each). Data are means ± s.e.m. compared by a two-tailed, unpaired *t*-test. **f**, As in **a**, except that dCas9-KRAB-MeCP2 is expressed instead. **g**,**h**, Compared to animals injected with dCas9-KRAB-MeCP2 plus NT sgRNA (*n* = 16), animals injected with dCas9-KRAB-MeCP2 plus *Arc* sgRNA (*n* = 12) showed impaired memory recall. Data are means ± s.e.m. compared either by two-way ANOVA with Sidak’s and Fisher’s post hoc tests (**g**) or by a one-tailed, unpaired *t*-test (**h**). **i**, As in D, except that dCas9-KRAB-MeCP2 is expressed instead. **j**, Quantification of **i**, showing impaired *Arc* expression in triple-positive neurons from the *Arc* sgRNA group compared to the NT sgRNA group (*n* = 3 each). Data are means ± SEM compared by a two-tailed, unpaired *t*-test. For all panels: NS, not significant; ^#^*P* ≤0.065, *P < 0.05 and ****P < 0.0001). See Supplementary Table 3 for summary statistics and *P* values.

**Extended Data Fig. 8:**
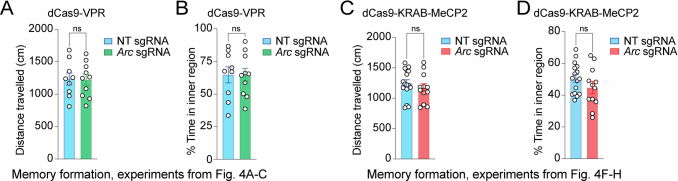
Epigenetic editing beyond memory malleability does not impact basal locomotion and anxiety levels. (**A-B**) For mice from the dCas9-VPR experiment of Fig.4a–c, displayed are (A) the average distance travelled and (B) the time spent in the inner region of the conditioning chamber during the three minutes habituation phase of the behavioral conditioning. For the NT or *Arc* sgRNA groups, n = 9 and 10, respectively. Data were compared by a two-tailed, unpaired t test (ns, not significant) and plotted as means ± SEM. (**C-D**) As in A-C, except that values referrer to the dCas9-KRAB-MeCP2 experiment of Fig.4f–h. For the NT or *Arc* sgRNA groups, n = 16 and 12, respectively. Data were compared by a two-tailed, unpaired t test (ns, not significant) and plotted as means ± SEM. For all figure panels, see Supplementary Table 3 for summary statistics and p-values.

Finally, we asked whether the epigenetic regulation of *Arc* could also have a mnemonic effect outside the initially labile phase of learning when newly encoded memories have become consolidated and are, by definition, less susceptible to change^25,26^. To this end, we deployed our double transgenic labeling system and stereotaxically delivered a TRE-dependent version of either dCas9-VPR or dCas9-KRAB-MeCP2 alongside the sgRNAs to the DG of cFos-Cre^ERT2^/rtTA^LSL^ mice. These mice were then trained on CFC, immediately after which they received an injection of 4-OHT and thus started to express rtTA in DG engram cells. At 4 days after CFC, that is, beyond the initially labile period after memory formation^27–29^, mice were put on DOX, which switched on the expression of the dCas9 components (Fig. 4a,d,f,i). Finally, another 3 days later, mice were tested for their memory retention. We found that dCas9-VPR plus *Arc* sgRNA mice displayed increased memory retention, which was accompanied by increased *Arc* expression in DG engram cells (Fig. 4b–e). Conversely, dCas9-KRAB-MeCP2 plus *Arc* sgRNA mice froze significantly less than the NT group, which was paralleled by reduced *Arc* mRNA levels (Fig. 4g–j). For both experiments, percentages of infected cells, locomotion and anxiety measurements did not differ between the *Arc* and NT sgRNA groups (Fig. 4e,j and Extended Data Fig. 8a–d). These findings indicate that the epigenetic editing of *Arc* within DG engram cells can bidirectionally alter memory expression even for fully consolidated memories.

## Discussion

Taken together, by engineering a spatiotemporally inducible CRISPR-based epigenetic editing system in engram cells, we found that the epigenetic makeup of a single locus is necessary and sufficient to regulate memory expression in reversible manner. From a neurobiological perspective, these experiments provide the hitherto finest-grained evidence of how epigenetic dynamics impact memory capacities: not only do epigenetic mechanisms accompany, but causally regulate memory formation. These findings mechanistically expand previous observational studies on how transcriptional^30^ and chromatin^18^ landscapes unfold over the timecourse of a memory, and reach beyond the interventional approaches that targeted the epigenetic machinery either pharmacologically or genetically^14,22,31,32^. With the caveat that we epigenetically interrogated only one genomic site in one brain region, in one memory task and only in male animals, these findings reveal chromatin alterations as functionally relevant mnemonics to regulate the behavioral expression of learnt information.

From a methodological perspective, this study illustrates how epigenetic editing tools, including the anti-CRISPR protein AcrIIA4, can be directed to designated cell populations in the adult mouse brain. Given its modular setup, similar experimental approaches may in future studies prove useful to better understand other phenotypes from the perspective of cell-type-specific and locus-resolved epigenetic regulation: within the field of memory research for disorders characterized by aberrant memory processing such as in traumatic memories or neurodegeneration; beyond the field of memory research, where epigenetic mnemonics have been proposed for drug-related memories^33^ as well as for stress-associated behaviors following childhood trauma^34^; and also outside the field of neuroscience, where cellular memories established during development^35^ following inflammation^36^ or after immune activation^37^ are known to be epigenetically encoded. Ultimately, such tools will therefore allow us to fully apprehend to what extent these epigenetic forms of cellular memory have been assimilated by the nervous system to store and express learnt information.

## Methods

All animals and procedures used in this study were approved by the Veterinary Office of the Federal Council of Switzerland under the animal experimentation license VD2808.2.

### sgRNA design and cloning

To design guide sequences for the CRISPR–dCas9 systems a total of 439 bp spanning the promoter of the mouse gene *Arc* were used as input for the CRISPROR program^38^. Five sgRNAs were selected and DNA oligonucleotides corresponding to the sgRNA sequences (Supplementary Table 2) were cloned individually into pLenti SpBsmBI sgRNA Hygro (Addgene 62205) containing an U6-driven sgRNA scaffold. Afterwards, the individual sgRNAs were multiplexed using NEBridge Golden Gate Assembly kit (NEB) with custom designed primers and cloned into the lentiviral pLVX-EF1a-GFP backbone. The sgRNA sequence against the bacterial *LacZ* gene was used as an NT control sgRNA as previously described^39^. The plasmids for dCas9-VPR and dCas9-KRAB-MeCP2 were generated by subcloning the lenti-EF1a-dCas9-VPR-Puro (Addgene 99373) and the lenti-SYN-dCas9-KRAB-MeCP2 (gift from J. Day) into the lentiviral pLVX backbone under control of either the CMV promoter (in vitro experiments in N2A cells) or the TRE (in vivo experiments). The dCas9-CBP constructs were obtained by replacing the VPR part with the CBP catalytic domain we previously characterized^40^. For the AcrIIA4 experiments, the pLVX-DIO-hSyn-dCas9-VPR plasmid was generated by subcloning the dCas9-VPR sequence into the pLVX-DIO-hSyn backbone; the pLVX-hU6-Arc sgRNAs-EF1a-GFP-TRE-mCherry-P2A-AcrII4A (in vitro experiments) and the pLVX-TRE-mCherry-P2A-AcrII4A (in vivo experiments) were generated starting from pHR-Ef1a-mCherry-P2A-AcrIIA4 (Addgene 125148); the pSIN-hPGK-rtTA plasmid was a gift from D. Trono. For a full list of the plasmids used, see Supplementary Table 2.

### Lentivirus production

Lentiviral vectors were produced as described previously^41^. HEK-293T cells (ATCC) were transfected using calcium phosphate with a second-generation packaging system composed of the pMD2G and psPAX2 vectors alongside the different pLVX vectors used in this study. After 4 days, medium was collected and centrifuged in an ultracentrifuge at 45,000*g* for 90 min at 4 °C. The pellet was resuspended in 0.5% bovine serum albumin (BSA)-PBS and viral titer determined using the HIV-1 p24 antigen ELISA kit (Zeptometrix Corp.). Injections were performed with 500 ng of each virus, which corresponded to a volume between 200 nl and 500 nl depending on the individual titer.

### Cell culture and transfection

N2A cells (ATCC), were cultured in Dulbecco’s Modified Eagle Medium GlutaMAX (Gibco) supplemented with a 1% penicillin-streptomycin solution (Gibco) and 10% fetal bovine serum (Gibco). Cells were transfected with the appropriate plasmids using Lipofectamine 2000 (Invitrogen) according to manufacturer’s instructions. To induce the expression of the AcrIIA4 construct, cells were treated with 1 μg ml^−1^ DOX.

### Immunocytochemistry

N2A cells were cultured on coverslips coated with poly-D-lysine (Corning) and transfected as described above. After 48 h, cells were fixed for 12 min at room temperature in a 4% paraformaldehyde (PFA), 4% sucrose PBS-based solution. Next, samples were incubated overnight at 4 °C with the primary antibody in a 0.3% Triton, 3% BSA, 10% serum PBS-based solution. The following day, cells were washed with PBS and incubated with the secondary antibody for 1 h at room temperature. Finally, coverslips were mounted on a slide with Vectashield–4′,6-diamidino-2-phenylindole (DAPI) and imaged with an Olympus Slide Scanner (Olympus VS120) at ×10 magnification. Antibodies used are listed in Supplementary Table 2.

### Immunoprecipitation assay

N2A cells treated as described above were resuspended in immunoprecipitation buffer (20 mM Tris-HCl (pH 7.5), 100 mM NaCl, 1 mM NaCl, 1 mM EDTA, 0.1% Triton X-100), shortly sonicated (five cycles 3 s on–off, Bandelin Sonopuls sonicator) and centrifuged for 10 min at 16,000*g* at 4 °C. Following a 1:3 dilution in 10 mM Tris-HCl (pH 7.5), 150 mM NaCl and 0.5 mM EDTA, lysates were incubated at 4 °C with either Flag or CBP antibody for 2 h, and then with G Dynabeads overnight. The day after, G Dynabeads were washed three times with dilution buffer and eluted in Laemmli buffer. For western blots, protein lysates were resolved in 7.5% SDS–polyacrylamide gels and transferred to nitrocellulose membranes. Proteins were visualized with horseradish peroxidase-conjugated secondary antibodies and ECL detection reagents (Amersham). Antibodies used are listed in Supplementary Table 2.

### RNA extraction and quantitative PCR

Total RNA was extracted using Trizol (Thermo Fisher Scientific), and cDNA synthesized using the SuperScript III First-Strand Synthesis kit (Invitrogen) according to the manufacturer’s instructions. All quantitative PCRs (qPCRs) were performed with the FAST SYBR Green Master Mix (Applied Biosystems). Each biological sample was loaded in technical triplicates and fluorescence acquisition carried out on a StepOnePlus Real-Time PCR System (Applied Biosystems). Calculations were performed using the ΔΔCt method and levels of Gapdh mRNA and Actin mRNA as reference genes. Primer sequences are listed in Supplementary Table 2.

### Chromatin immunoprecipitation

Samples for chromatin immunoprecipitation (ChIP)–qPCR were obtained as described previously with minor adaptations^42^. N2A cells were crosslinked using 1% formaldehyde for 10 min at room temperature, followed by lysis in 5 mM HEPES (pH 8), 85 mM KCl, 0.5% NP-40. After centrifugation, nuclei were washed in 5 mM HEPES (pH 8), 85 mM KCl and then sonicated in 1 ml of 50 mM Tris-HCl (pH 8), 10 mM EDTA, 1% SDS (Covaris LE220 sonicator). The resulting chromatin was precleared with A or G Dynabeads (Invitrogen) and incubated overnight with antibodies in immunoprecipitation (IP) buffer (50 mM HEPES (pH 7.5), 1% Triton X-100, 150 mM NaCl). Next, A or G Dynabeads were added for 6 h, followed by eight washes: the first three in IP buffer plus 0.1% sodium deoxycholate, 0.1% SDS and 1 mM EDTA; the second three in the same buffer but with 500 mM NaCl; then one wash in 10 mM Tris-HCl pH 8, 250 mM LiCl, 1 mM EDTA, 1% NP-40 and 0.5% sodium deoxycholate; and a final wash in 10 mM Tris-HCl pH 8, 1 mM EDTA. Chromatin was then eluted in 1% SDS, 0.1 M NaHCO_3_ and incubated at 65 °C overnight for decrosslinking plus Proteinase K digestion, before clean up using the QiaQuick PCR purification kit (Qiagen). qPCR was carried out as for gene expression analysis. Values for each IP sample were normalized relative to corresponding input chromatin for the same treatment. Primer sequences and antibodies are listed in Supplementary Table 2.

### ChIP–seq library preparation and analysis

Libraries from samples prepared as described above were generated using the NEBNext Ultra II DNA Library Preparation Kit (NEB) according to manufacturer’s instructions. Samples were multiplexed and 75-bp paired-end reads generated on a Nextseq500 (Illumina). After reads were trimmed for NEBNext Ultra II DNA (TruSeq) adapters, the FastQ files were demultiplexed using bclconvert (v.3.9.3, Illumina) and aligned to the mm10 genome using bowtie2 (v.2.4.5) in paired-end mode using default parameters. The R library Csaw (v.1.30.1) was used for peak calling, with peaks being considered nonsignificant for regions that had less than a threefold enrichment compared to their 2-kb neighborhood, and reads were normalized using the locally estimated scatterplot smoothing (loess) algorithm. Peaks were assigned to the nearest promoter with the distanceToNearest function from GenomicRanges, and a differential enrichment analysis performed using EdgeR (v.3.38.4). The threshold for significance was set at log fold change (FC) > 2 and *P* value < 0.05. When assigning peaks to genomic features promoter regions were defined as 2 kb upstream and 200 bp downstream of the gene transcription start site (TSS). ChIP–seq data were visualized using the Integrative Genomics Viewer (v.2.16.0). See Supplementary Table 1 for results summary.

### Animals

cFos-tTA mice were bred inhouse from the original Jackson Laboratory (JAX strain 018306) on a C57Bl/6JR background. Animals were group-housed in a 12-h light/dark cycle with water and food available ad libitum. DOX (0.2 mg ml^−1^) was given orally through the water supply beginning at least 7 days before the start of the experiment, and only ceased 3 days before the session where the tagging window was desired. DOX was provided back to the animals as soon as the tagging window was no longer needed. Double transgenic cFos-Cre^ERT2^/R26-CAG-rtTA^LSL^ animals were generated inhouse by crossing the original JAX strains 030323 and 029617. cFos-Cre^ERT2^/R26-CAG-rtTA^LSL^ mice were injected with tamoxifen (4-OHT, Sigma-Aldrich, 50 mg kg^−1^) immediately after fear conditioning. Powdered tamoxifen was dissolved in ethanol 100% at a concentration of 20 mg ml^−1^ and stored at −20 °C. On the day of the experiment, tamoxifen was redissolved by shaking at 37 °C, then two volumes of corn oil were added and ethanol was evaporated shaking at 37 °C for a final concentration at 10 mg ml^−1^. Tamoxifen was kept at 37 °C until injection. Mice were between 8 weeks and 13 weeks old at the start of the experiments, and all were male. All behavioral procedures were performed between 1 pm and 5 pm local time and animals were assigned randomly to experimental groups.

### Behavioral procedures

CFC experiments were performed using a TSE Multi Conditioning System. CFC encoding consisted of a first 3-min exploration phase, followed by 2-s long foot shocks spaced by 28 s. The number of repetitions of the pause-shock stage as well as the electrical current varied depending on the experiment (as illustrated in figures). After the last shock, the animal was left in the chamber for an additional 15 s and brought back to its home cage. The recall phase consisted of a 3-min exposure to the same context, without any shock. The time after encoding at which recall took place varied depending on the experiment. The movement of the animals was measured automatically using an infrared beam cut detection system (TSE Systems) and freezing calculated as the absence of movement for more than 500 ms. Open field experiments consisted of a 15-min long session during which the animals were left exploring an open arena (72 × 72 cm). Sessions were analyzed using Ethovision System (Noldus).

### Virus injections

Surgeries were performed as described with minor adaptations^43^. Before surgery, mice were injected intraperitoneally with an anesthetic mix of fentanyl (0.05 mg kg^−1^), midazolam (5 mg kg^−1^), and metedomidin (0.5 mg kg^−1^), followed by a subcutaneous injection of an analgesic mix (lidocaine 6 mg kg^−1^ and bupivacaine 2.5 mg kg^−1^) at the surgery site. Animals were then placed on a stereotaxic micromanipulator frame (Kopf Instruments), skin disinfected with betadine and opened with a scalpel. To target the DG, holes were drilled in the skull with a 30-gauge drill bit at ±1.3 mm medio-lateral, −2.0 mm anterior–posterior. A virus-loaded micropipette (BLAUBRAND, intraMARK, tip diameter 10–20 µm) was lowered to −2.0 mm and 500 ng of viral particles were injected bilaterally at a rate of 0.2 µl min^−1^. After 5 min, the micropipette was raised 20 µm from the target for 1 min to prevent backflow and allow diffusion, then removed at 10 µm s^−1^. After the injections, the skin was sutured and atipamezol (2.5 mg kg^−1^) administered intraperitoneally for anesthesia reversal. Postsurgery, mice were moved to a clean cage on a heated pad until regaining mobility, then returned to their home cage with paracetamol provided in the drinking water for 1 week (500 mg per 250 ml). Surgery efficiency was verified by visualizing the GFP signal and misinjected brains were excluded from further analyses.

### Nuclei preparation for scATAC-seq and single-cell RNA sequencing

Mice were euthanized 8 min after the recall session and the DG microdissected, flash-frozen and stored at −80 °C. Nuclei were extracted as described previously with minor adaptations^44^. To obtain a sufficient number of nuclei for scATAC-seq, the DGs from three animals were pulled for each condition. For single-cell RNA sequencing (scRNA-seq) each mouse was instead processed individually. All steps were performed at 4 °C. Brain tissue was first homogenized in homogenization buffer (250 mM sucrose, 25 mM KCl, 5 mM MgCl_2_, 10 mM Tris-HCl, pH 7.4, 1 mM dithiothreitol, 1× protease inhibitor, 1 U μl^−1^ RiboLock RNase inhibitor, 0.1% NP-40, in H_2_O), filtered through a 40-μm cell strainer and washed with nuclei isolation medium (without NP-40). Samples were centrifuged for 8 min at 1,000*g*, and nuclei resuspended in PBS/BSA storage solution (PBS 1×, 5 mM MgCl_2_, 0.5% BSA, 1 mM dithiothreitol in H_2_O) followed by filtering through 35-μm strainers. FANS was performed with a Sony SH800 sorter using a 100-μm nozzle and nuclei sorted on the basis of the GFP signal. For scATAC-seq, sorted nuclei were incubated for 2 min in permeabilization buffer (nuclei isolation medium plus 0.01% Tween, 0.001% Digitonin, 0.01% NP-40, 0.5% BSA), then washed in nuclei isolation buffer plus 0.5% BSA, resuspended in diluted nuclei buffer (Chromium nuclei isolation kit, 10x Genomics) and finally processed for GEMs generation using Chromium X and the ATAC NextGEM v.2 kit (10x Genomics). For scRNA-seq, sorted nuclei were fixed using the Evercode Low Input Fixation kit (Parse Biosciences) according to the manufacturer’s instructions and stored at −80 °C until libraries preparation.

### scATAC-seq and scRNA-seq library preparation and analyses

The individual scATAC-seq libraries matching each condition (*Arc* or NT sgRNA) were pooled and 65-bp paired-end reads were generated on an Aviti platform (Element Biosciences), with a mean depth of ~200,000 reads per cell. Raw files were aligned and features were counted using default parameters for Cellranger-atac count^45^. The R tools Signac^46^ and Seurat^47^ with default parameters were used for downstream processing. Cells were maintained if they had between 300 and 75,000 unique molecular identifier (UMI) counts and genes were kept if they had greater than 30 reads, a TSS enrichment of more than two and a nuclear signal less than four. Cell types were defined by using the values calculated in the gene activity matrix with MapMyCells^48^. Cas9-positive cells were defined by performing a targeted PCR on each library, sequencing and then searching sequences matching the 3’ end of the Cas9 gene and extracting the cell barcode. Cells were considered Cas9 positive if the barcode has at least one Cas9 sequence hit in the PCR analysis or the initial library sequencing run. For the dCas9-positive cells across experimental conditions the normalized reads count at the TSS location of genes of interest were extracted and plotted as violin plots. For scRNA-seq, individual libraries from four *Arc* sgRNA and five NT sgRNA mice were prepared using the Evercode WT v.3 kit (Parse Biosciences) before being combined into eight sublibraries according to the manufacturer’s instructions. Sublibraries were sequenced on an Aviti platform (Element Biosciences) with a reads configuration of 150-bp paired-end and at depth of ~100 million reads per sample. Next, raw files were aligned with Parse Biosciences Trailmaker (v.1.4.1) to a modified version of the mouse genome (mm10) that includes GFP and Cas9m4 sequences. Seurat^47^ was used to filter for cells that had between 300 and 10,000 expressed genes, between 500 and 100,000 UMI counts and fewer than 1% mitochondrial reads. Note, only genes that were present in at least 20 cells were considered for further analysis. Each library was then separately normalized using SCTransform^49^ on the top 3,000 features before all libraries were integrated together. MapMyCells^48^ was used to identify cell types and Cas9-positive cells were defined as being any cells with at least 1 UMI count in the Cas9 sequence region. Finally, the Seurat^47^ command FindMarkers() was used to define differentially expressed genes between cells coming from *Arc* and NT samples in each cell type, with a *P* value ≤ 0.01 as cut-off. A summary of the scATAC-seq and scRNA-seq results is provided in Supplementary Table 1.

### Histology and RNAscope

Eight minutes after the last behavioral test, animals were anesthetized with pentobarbital (150 mg kg^−1^) and perfused transcardially with first PBS and then 4% PFA in PBS. Brains were extracted, postfixed overnight in 4% PFA, transferred in a cryoprotectant solution (30% sucrose in PBS) for at least 48 h, and frozen at −80 °C. Sections of 20 μm were cut using a cryostat, and stored at −20 °C in an antifreeze solution (30% ethylene glycol, 15% sucrose, 0.02% azide in PBS). RNAscope followed by immunohistochemistry was performed using the RNAscope Multiplex Fluorescent v.2 kit (ACD Bio) according to the manufacturer’s instructions. Probes of interest (Supplementary Table 2) were hybridized for 2 h at 40 °C and the signal was amplified through AMP1, AMP2 and AMP3 probes before being developed using the appropriate horseradish peroxidase signal. Finally, the presence of GFP was revealed using a standard immunohistochemistry protocol. Slices were blocked 30 min in 1% BSA-PBS and incubated overnight at 4 °C with the anti-GFP primary antibody (Supplementary Table 2) in 1% BSA-PBS. The next day, the secondary antibody (Supplementary Table 2) was applied for 1 h at room temperature and nuclei were stained with Hoechst dye. The immunohistochemistry experiment of Extended Data Fig. 1 was performed as described previously^43^ using the primary and secondary antibodies listed in Supplementary Table 2.

### Confocal images acquisition and analysis

An Upright Leica DM6 CS laser scanning confocal microscope was used to acquire images of the DG for each brain slice. All slides were acquired at ×63 magnification with a resolution of 512 × 512, speed of 400 Hz, airy unit between 0.3 and 0.4 a.u. and line averaging of three. Channels were acquired from the longest wavelength to the shortest wavelength and acquired one at a time to avoid leakage between the channels with spectrum overlaps assessed with BD spectrum viewer (BD Biosciences). A tilescan of the acquired area was then stitched within the Leica LAS-X software. Images were analyzed using QuPath (v.0.2.3 to v.0.4.3)^50^. For each confocal image of a single DG, detected nuclei were first classified as part of the DG granular cell layer or not using a machine-learning based algorithm developed inhouse starting from the StarDist library. Next, nuclei were defined as positive for each given channel if their mean signal was higher than a manually preset threshold of detection. Finally, measurements from all detected nuclei were exported and analyzed using Phyton scripts. To compute *Arc* expression levels for the triple-positive nuclei (sgRNA^+^, *dCas9*^+^, *Arc*^+^), the mean intensity value corresponding to the *Arc* probe signal for each nucleus in a given DG was extracted and averaged to obtain a single value per acquired image. Overall, a total of four images was analyzed for each mouse, and three to four mice per group were compared in each experiment.

### Statistics and reproducibility

Statistical analysis was performed in Prism 9 (GraphPad) and all data are displayed as mean ± s.e.m. For in vivo experiments, no statistical methods were used to predetermine sample sizes, but the number of animals used in each experiment is similar to those reported in previously published engram studies. Data distribution was assumed to be normal, although this was not formally tested. Animals were assigned randomly to experimental groups and age matched. For in vitro experiments, at least three independent experiments were performed for statistical analysis unless otherwise specified in the figure legends. The statistical test used and the definition of *n* are described in the figure legends. Statistical analysis details for each figure are reported in Supplementary Table 3.

### Reporting summary

Further information on research design is available in the Nature Portfolio Reporting Summary linked to this article.

## Online content

Any methods, additional references, Nature Portfolio reporting summaries, source data, extended data, supplementary information, acknowledgements, peer review information; details of author contributions and competing interests; and statements of data and code availability are available at 10.1038/s41588-025-02368-y.

## Supplementary information

Reporting Summary

Supplementary Table 1 Analysis results for the dCas9-KRAB-MeCP2 ChIP–seq, scATAC-seq and scRNA-seq experiments.

Supplementary Table 2 List of materials, reagents and software used in this study.

Supplementary Table 3 Statistics summary for all experiments in this study.

## Source data

Source Data Fig. 2 Unprocessed western blots for Fig. 2j.

## Data Availability

The list of materials, reagents and software used in this study is provided in Supplementary Table 2. ChIP–seq, scATAC-seq and scRNA-seq data have been deposited in the Gene Expression Omnibus (GEO) database, https://www.ncbi.nlm.nih.gov/geo (accession no. GSE299742), all other data are available in the main text or in the Supplementary Information. Source data are provided with this paper.

## Code availability

Custom codes developed in this study are available via Zenodo at 10.5281/zenodo.16834059 (ref. ^51^).
