# Supplementary material for: Cell-type- and locus-specific epigenetic editing of memory expression
Source: Nat Genet. 2025 Oct 29;57(11):2661–8. doi: 10.1038/s41588-025-02368-y (PMC12597831; doi:10.1038/s41588-025-02368-y)
Supplement: Supplementary file 1 — Reporting Summary [file 41588_2025_2368_MOESM1_ESM.pdf]

nature portfolio

Corresponding author(s): Johannes Gräff

Last updated by author(s): Aug 12, 2025

Reporting Summary

Nature Portfolio wishes to improve the reproducibility of the work that we publish. This form provides structure for consistency and transparency in reporting. For further information on Nature Portfolio policies, see our Editorial Policies and the Editorial Policy Checklist.

Statistics

For all statistical analyses, confirm that the following items are present in the figure legend, table legend, main text, or Methods section.

n/a	Confirmed	
☐	☒	The exact sample size (*n*) for each experimental group/condition, given as a discrete number and unit of measurement
☐	☒	A statement on whether measurements were taken from distinct samples or whether the same sample was measured repeatedly
☐	☒	The statistical test(s) used AND whether they are one- or two-sided<br>*Only common tests should be described solely by name; describe more complex techniques in the Methods section.*
☐	☒	A description of all covariates tested
☐	☒	A description of any assumptions or corrections, such as tests of normality and adjustment for multiple comparisons
☐	☒	A full description of the statistical parameters including central tendency (e.g. means) or other basic estimates (e.g. regression coefficient) AND variation (e.g. standard deviation) or associated estimates of uncertainty (e.g. confidence intervals)
☐	☒	For null hypothesis testing, the test statistic (e.g. *F*, *t*, *r*) with confidence intervals, effect sizes, degrees of freedom and *P* value noted<br>*Give P values as exact values whenever suitable.*
☒	☐	For Bayesian analysis, information on the choice of priors and Markov chain Monte Carlo settings
☒	☐	For hierarchical and complex designs, identification of the appropriate level for tests and full reporting of outcomes
☒	☐	Estimates of effect sizes (e.g. Cohen's *d*, Pearson's *r*), indicating how they were calculated

Our web collection on statistics for biologists contains articles on many of the points above.

Software and code

Policy information about availability of computer code

Data collection	TSE, Multi-conditioning System, automatic freezing detection; StepOne Software V2.3 by Applied Biosystems, Real-Time PCR data acquisition; Leica LAS-X , confocal images acquisition.
Data analysis	QuPath 0.4.3, image analysis; GaphPad Prism 9, data plotting and statistical analysis; IGV 2.16.0 ChIP-seq tracks visualization; EdgeR, ChIP-seq differential enrichment analysis; Parse Biosciences TrailmakerTM (v1.4.1), scRNA-seq allignement; Signac and Seurat for end-to-end scATAC and scRNA-seq data analysis. Custom codes developed in this study have been deposited in the Zenodo repository and are available at https://doi.org/10.5281/zenodo.16834060.

For manuscripts utilizing custom algorithms or software that are central to the research but not yet described in published literature, software must be made available to editors and reviewers. We strongly encourage code deposition in a community repository (e.g. GitHub). See the Nature Portfolio guidelines for submitting code & software for further information.

Data

Policy information about availability of data

All manuscripts must include a data availability statement. This statement should provide the following information, where applicable:

- Accession codes, unique identifiers, or web links for publicly available datasets
- A description of any restrictions on data availability
- For clinical datasets or third party data, please ensure that the statement adheres to our policy

Raw data are available in the Gene Expression Omnibus (GEO) database, https://www.ncbi.nlm.nih.gov/geo (accession no. GSE299742)

Research involving human participants, their data, or biological material

Policy information about studies with human participants or human data. See also policy information about sex, gender (identity/presentation), and sexual orientation and race, ethnicity and racism.

Reporting on sex and gender	*Use the terms sex (biological attribute) and gender (shaped by social and cultural circumstances) carefully in order to avoid confusing both terms. Indicate if findings apply to only one sex or gender; describe whether sex and gender were considered in study design; whether sex and/or gender was determined based on self-reporting or assigned and methods used. Provide in the source data disaggregated sex and gender data, where this information has been collected, and if consent has been obtained for sharing of individual-level data; provide overall numbers in this Reporting Summary. Please state if this information has not been collected. Report sex- and gender-based analyses where performed, justify reasons for lack of sex- and gender-based analysis.*
Reporting on race, ethnicity, or other socially relevant groupings	*Please specify the socially constructed or socially relevant categorization variable(s) used in your manuscript and explain why they were used. Please note that such variables should not be used as proxies for other socially constructed/relevant variables (for example, race or ethnicity should not be used as a proxy for socioeconomic status). Provide clear definitions of the relevant terms used, how they were provided (by the participants/respondents, the researchers, or third parties), and the method(s) used to classify people into the different categories (e.g. self-report, census or administrative data, social media data, etc.) Please provide details about how you controlled for confounding variables in your analyses.*
Population characteristics	*Describe the covariate-relevant population characteristics of the human research participants (e.g. age, genotypic information, past and current diagnosis and treatment categories). If you filled out the behavioural & social sciences study design questions and have nothing to add here, write "See above."*
Recruitment	*Describe how participants were recruited. Outline any potential self-selection bias or other biases that may be present and how these are likely to impact results.*
Ethics oversight	*Identify the organization(s) that approved the study protocol.*

Note that full information on the approval of the study protocol must also be provided in the manuscript.

Field-specific reporting

Please select the one below that is the best fit for your research. If you are not sure, read the appropriate sections before making your selection.

☒ Life sciences ☐ Behavioural & social sciences ☐ Ecological, evolutionary & environmental sciences

For a reference copy of the document with all sections, see nature.com/documents/nr-reporting-summary-flat.pdf

Life sciences study design

All studies must disclose on these points even when the disclosure is negative.

Sample size	No statistical methods were used to pre-determine sample sizes. Sample sizes used were based on previous publications performing similar experiments (Khalaf et. al 2018; Silva et al., 2021)
Data exclusions	Animal were excluded from further experimentation or data analysis if they displayed misplaced stereotaxic injections or poor viral expression
Replication	All experiments were independently replicated in at least 2 cohorts of animals (n number of each experimental group is reported in every figure legend). All in vitro experiments were independently replicated 3 times.
Randomization	Mice were raised and housed in the same conditions and randomly allocated to the different experimental groups.
Blinding	The investigators were blinded to the experimental group for sample processing, image analysis and behavioral analysis.

Reporting for specific materials, systems and methods

We require information from authors about some types of materials, experimental systems and methods used in many studies. Here, indicate whether each material, system or method listed is relevant to your study. If you are not sure if a list item applies to your research, read the appropriate section before selecting a response.

Materials & experimental systems

n/a	Involved in the study
☐	☒ Antibodies
☐	☒ Eukaryotic cell lines
☒	☐ Palaeontology and archaeology
☐	☒ Animals and other organisms
☒	☐ Clinical data
☒	☐ Dual use research of concern
☒	☐ Plants

Methods

n/a	Involved in the study
☐	☒ ChIP-seq
☒	☐ Flow cytometry
☒	☐ MRI-based neuroimaging

Antibodies

Antibodies used	H3K27ac, Abcam 4729 (ChIP,1 ug/1 million cells); Histone H3, Abcam 1791 (ChIP,1 ug/1 million cells); GFP, Abcam 6673 (IHC, 1:400); Flag, Sigma F1804 (IF(1:500), ChIP (1 ug/1 million cells), IP (0.75 ug/1 million cells), WB (1:1000)); CBP Santa Cruz C1 sc7300 (IP (0.75 ug/1 million cells), WB (1:400)); Arc, Synaptic System 156 003 (IHC, 1:1000); Fos, Synaptic System 226 308 (IHC, 1:4000)
Validation	The specificity of the antibody have been validated by the manufacturers. A list of scientific publications about antibody validation can be found on the manufacturers website. References list for anti H3K27ac, Abcam 4729 can be found at https://www.abcam.com/products/primary-antibodies/histone-h3-acetyl-k27-antibody-chip-grade-ab4729.html. References list for anti Histone H3, Abcam 1791 can be found at https://www.abcam.com/en-at/products/primary-antibodies/histone-h3-antibody-nuclear-marker-and-chip-grade-ab1791. References list for anti GFP, Abcam 6673 can be found at https://www.abcam.com/products/primary-antibodies/gfp-antibody-ab6673.html. References list for anti Flag, Sigma F1804 can be found at https://www.sigmaaldrich.com/CH/en/product/sigma/f1804. References list for anti CBP, Santa Cruz C1 sc7300 can be found at https://datasheets.scbt.com/sc-7300.pdf. References list for anti Arc, Synaptic System 156 003 can be found at https://www.sysy.com/product/156003. References list for anti Fos, Synaptic System 226 308 can be found at https://sysy.com/product/226308.

Eukaryotic cell lines

Policy information about cell lines and Sex and Gender in Research

Cell line source(s)	N2A and HEK293T cells were obtained from ATCC
Authentication	None
Mycoplasma contamination	cells tested negative for mycoplasma contamination
Commonly misidentified lines (See ICLAC register)	none

Animals and other research organisms

Policy information about studies involving animals; ARRIVE guidelines recommended for reporting animal research, and Sex and Gender in Research

Laboratory animals	Mus musculus; cFos-tTA mice were bred in house from the original JAX strain #018306 on a C57Bl/6JR background; cFos-CreERT2/R26-CAG-rtTALSL animals were generated in house by crossing the original JAX strains #030323 and #029617. All mice were between 8-13 weeks old at the start of the experiments
Wild animals	The study did not involve wild animals
Reporting on sex	Only male mice were involved in this study
Field-collected samples	The study did not involve samples collected from the field
Ethics oversight	The federal Food Safety and Veterinary Office of the Federal Council of Switzerland approved the animals license VD2808.2 for the experiments performed in this study.

Note that full information on the approval of the study protocol must also be provided in the manuscript.

Plants

Seed stocks	no plants were involved in this study.
Novel plant genotypes	no plants were involved in this study.
Authentication	no plants were involved in this study.

ChIP-seq

Data deposition

☒ Confirm that both raw and final processed data have been deposited in a public database such as GEO.

☒ Confirm that you have deposited or provided access to graph files (e.g. BED files) for the called peaks.

Data access links<br>*May remain private before publication.*	Link has been requested at the GEO repository.
Files in database submission	fastq and bigwig files for 8 samples (2 replicates each of Arc/NT IP or input)
Genome browser session<br>(e.g. UCSC)	bigwig files are provided in the GEO repository for viewing in IGV of UCSC

Methodology

Replicates	2 biological replicates for condition
Sequencing depth	approximately 40 million paired end reads
Antibodies	Flag, Sigma F1804
Peak calling parameters	The R library csaw (v.1.30.1) was used for peak calling, with peaks being considered non-significant for regions that had less than a three-fold enrichment compared to their 2 kb neighbourhood, and reads were normalized using the locally estimated scatterplot smoothing (loess) algorithm.
Data quality	Approximately 60-80% of reads mapped for flag samples and 95% of reads mapped to the mouse gened for input samples. Average fragment lengths were approximately 200 bp for all samples. PCA analysis shows that input samples cluster and replicates of IP samples cluster together.
Software	Reads were trimmed for NEBNext Ultra II DNA (TruSeq) adaptors. FastQ files were demultiplexed using bclconvert (v3.9.3, Illumina) and aligned with bowtie2 (v2.4.5). The R library csaw (v.1.30.1) was used for peak calling. DE analysis was performed with EdgeR (v3.38.4).
