# Supplementary material for: Cell-type- and locus-specific epigenetic editing of memory expression
Source: Nat Genet. 2025 Oct 29;57(11):2661–8. doi: 10.1038/s41588-025-02368-y (PMC12597831; doi:10.1038/s41588-025-02368-y)
Supplement: Supplementary file 5 — Unprocessed western blots for Fig. 2j. [file 41588_2025_2368_MOESM5_ESM.pdf]

Figure 2J, blot for FLAG on FLAG IP samples

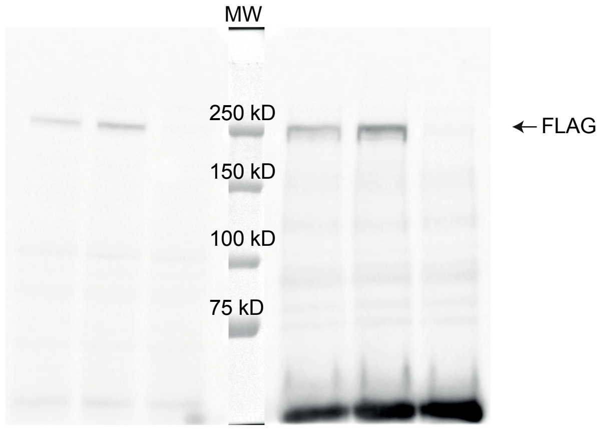

Figure 2J, blot for CBP on FLAG IP samples

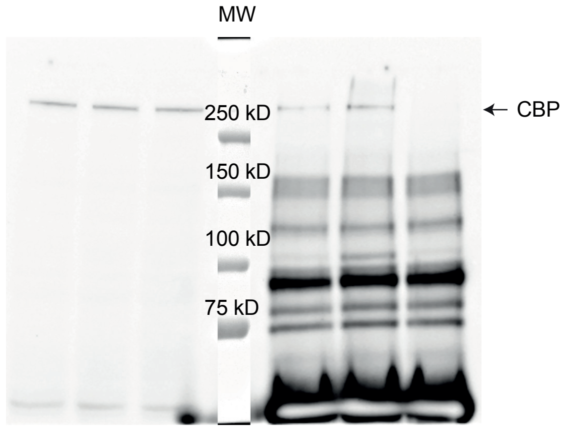

Figure 2J, blot for FLAG on CBP IP samples

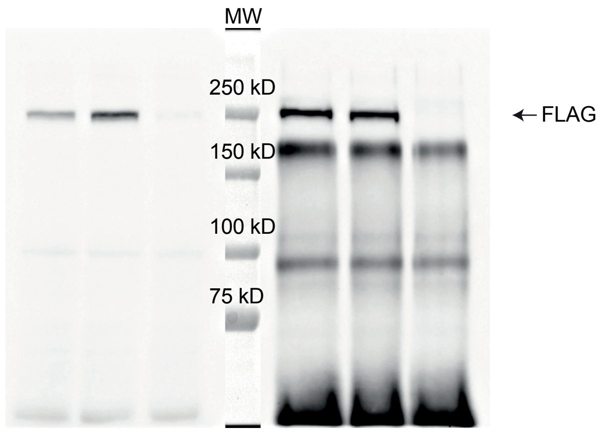

Figure 2J, blot for CBP on CBP IP samples

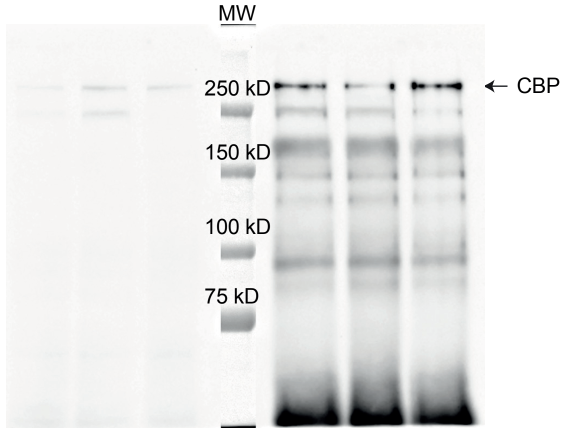

Coda et al. Source Data file 1
